# The role of cannabis in bipolar disorder relapse: a prospective study of hospital acute readmissions

**DOI:** 10.1192/j.eurpsy.2024.906

**Published:** 2024-08-27

**Authors:** L. Olivier, A. Giménez, H. Andreu Gracia, L. Bueno, Ó. De Juan Viladegut, T. M. Fernández, I. Ochandiano, S. Salmerón, L. Bracco, L. Tardón Senabre, I. Pacchiarotti

**Affiliations:** ^1^Institut de Neurociències, Hospital Clínic de Barcelona, Barcelona, Spain; ^2^Department of Pathophysiology and Transplantation de Neurociències, University of Milan, Milan, Italy; ^3^Bipolar Disorder Unit, Hospital Clínic de Barcelona, Barcelona, Spain

## Abstract

**Introduction:**

With the rapid changes of attitude, investigation and legislation around cannabis and its subproducts in the Western world, there is a need to profoundly examine the consequences of its use in the general population and, specifically, in people affected by mental disorders. There is a clear relationship between cannabis use and psychosis, but there is also growing evidence of its relationship with manic episodes (Sideli et al, 2019).

A systematic review published by the CANMAT Task Force in 2022 examined again the relationship between cannabis use and bipolar disorder (BD), establishing association with worsened course and functioning of BD in frequent users (Tourjman et al., 2023). On the other hand, some recent papers have highlighted the role of the endocannabinoid system (ECS) in BD, suggesting even possible beneficial effects, mainly through the CB2 receptor (Arjmand et al, 2019).

**Objectives:**

To describe the impact of cannabis in the psychiatric readmission in BD and to approach the differences in course in cannabis users with regards to non-users.

**Methods:**

We conducted a prospective cohort study including the patients admitted to our acute psychiatric unit with the diagnosis of manic or mixed episode during the period between 2015 and 2019 (including patients with one of both final diagnosis: BD or schizoaffective disorder). We established a follow-up of 3 years from the date of admission in which hospital readmissions are examined.

**Results:**

The study, which included 309 patients, concluded that cannabis users were admitted and had the first episode at a younger age (p=0.005), a higher percentage of them did not have a previous diagnosis (p=0.026) nor a previous history of mental health issues (p=0.019) and it was more likely to be their first admission (p=0.011) and to suffer psychotic symptoms (p=0.002).

As of treatment, the results were statistically significant regarding the fact that a lower proportion of patients had received previous psychiatric treatment (p=0.004) and previous electroconvulsive therapy (p=0.003). There was a higher chance of them being non-adherent with medication (p<0.001) and to be administered extended-release antipsychotic treatment during admission (p<0.001).

The study did not find a statistically significant relationship with cannabis use and a higher rate of readmission in the 3 years of follow-up.

**Conclusions:**

Although a higher relapse rate could not be proven in our study, other previously identified factors related to a worse illness course (Sajatovic et al., 2009) did show a significant association with cannabis use, which could lead to one suggesting that our results are compatible with the actual evidence and that cannabis products are detrimental to people who suffer from BD and schizoaffective disorder.

**Disclosure of Interest:**

None Declared

